# Translocation of Phthalates From Food Packaging Materials Into Minced Beef

**DOI:** 10.3389/fnut.2021.813553

**Published:** 2022-01-20

**Authors:** Denis Baranenko, Mohamed Said Boulkrane, Irina Borisova, Bazhena Astafyeva, Weihong Lu, A. M. Abd El-Aty

**Affiliations:** ^1^International Research Centre “Biotechnologies of the Third Millennium”, Faculty of Biotechnologies (BioTech), ITMO University, Saint-Petersburg, Russia; ^2^Institute of Extreme Environment Nutrition and Protection, Harbin Institute of Technology, Harbin, China; ^3^Department of Pharmacology, Faculty of Veterinary Medicine, Cairo University, Giza, Egypt; ^4^Department of Medical Pharmacology, Medical Faculty, Ataturk University, Erzurum, Turkey

**Keywords:** xenobiotics, toxicity, GC-MS, meat, animal raw materials

## Abstract

There has been increased concern regarding the potential human health risks associated with exposure to phthalates. Research indicates that food intake is the most critical exposure pathway for phthalates. This study aimed to investigate packaged beef samples for the presence of dimethyl terephthalate (DMTP), di-n-butyl phthalate (DnBP), and diisooctyl phthalate (DiOP) and to assess their translocation from the common form of food packaging procured from various Saint-Petersburg and Leningrad region shops. The packaging samples include paper and different types of plastic. Phthalates were extracted by dichloromethane and analyzed by gas chromatography coupled with mass spectrometry (GC-MS). While DnBP had the highest mean values in beef from 34.5 to 378.5 μg·kg^−1^, DiOP displayed the lowest mean values from LOD to 37 μg·kg^−1^. The larger contact area and the presence of distributed fat on the surface of the minced meat resulted in significantly higher phthalate translocation than beef slices. Further, DMTP was not detected in any samples. However, the examined food packages do not meet the requirements of Russian, EU and USA legislation, as DnBP migrates to meat. Calculated maximum DnBP daily intake of 0.167 μg·kg^−1^·day^−1^ for chilled minced beef in vacuum packaging did not exceed tolerable daily intake (TDI) level. The most alarming results are concerning the phthalates presence in beef farmed in the Leningrad region and not subjected to any plastic packaging. A full-scale study is warranted to determine the pathways and sources of phthalates migration in the food chain.

## Introduction

Over the past decades, a vast number of studies related to the existence of phthalate compounds in packaging materials and food have been published (1–4). Phthalates (diesters of ortho phthalic acid) are organic chemicals that are commonly used as plasticizers to make plastic polymers softer and more flexible. Additionally, they are used to produce lacquers and printing inks as additives to improve the flexibility, surface adhesion, color, elasticity, and wrinkle resistance to manufacturing adhesives, solvents, waxes, pharmaceuticals, and cosmetics insecticides, and packaging from regenerated cellulose (5).

The presence of phthalates in food poses a significant concern regarding their impact on human health (6). Following exposure and uptake, phthalates are rapidly metabolized by hydrolysis followed by conjugation (7). The first phase, which occurred in the intestine and parenchyma and catalyzed by esterases and lipases, the diester phthalate is hydrolyzed into the monoester phthalate (8). Monoester phthalates are more bioactive than non-hydrolyzed diester phthalates, as shown in *in vitro* and *in vivo* studies. Short-branched phthalates are mainly excreted as monoester phthalates *via* urine. In contrast, long-branched compounds are further biotransformed through hydroxylation and oxidation and then excreted in feces and urine as phase II conjugated compounds (9). Phthalates were also found in breast milk and amniotic fluid (10).

It was reported that exposure to phthalates and their metabolites might inadvertently be disrupting the endocrine system, act as carcinogens, and negatively impact the reproductive system (11). Some phthalates may have anti-androgenic and cause reproductive and developmental toxicity in animals (12). Researches demonstrated that phthalates could adversely affect the male reproductive system, causing asthma, rhinitis, and eczema in children, in addition to their impacts on metabolism and neurological development (13). Neurotoxicity, infertility, respiratory symptoms, epigenetic, immune and metabolic abnormalities are other endpoints of target organ toxicity caused by phthalates (14).

People are mainly exposed to phthalates from food, water, air, and consumer products, including building materials, household furnishings, clothing, cosmetics, pharmaceuticals, nutritional supplements, medical devices, dentures, children's toys, glow sticks, modeling clay, food packaging, automobiles, lubricants, waxes, cleaning materials, and insecticides (15). The routes of phthalate exposure ranked by their importance include food intake, dust ingestion, and indoor air inhalation (16). Mechanical stress or high temperature can cause chemical migration from packaging materials to food or drink (17). Phthalates are readily leached from products and released to the environment because of their weak bonds with bridging substrate (11).

In addition to the direct effect of phthalates through food items, their migration to the soil, groundwater, and dust can pose a significant risk to human health and/or sensitive environmental receptors. Plastics account for 25% of municipal solid waste (18). The most rational way for treating plastic is separate collection and recycling as it gives the possibilities for environmentally friendly management of every municipal solid waste stream (19). Even in this case, plastic materials should be handled, treated, and disposed of safely (20). In the case of landfilling of plastic materials, the impact of phthalates on environmental quality is associated with certain long-term related soil and groundwater contamination problems (21).

European Union has set limits and regulations for the content of chemical compounds in packaging. European Food Safety Authority has specified tolerable daily intakes (TDIs) of 0.01 mg × kg^−1^ bw for di-n-butyl phthalate (DnBP), 0.05 mg × kg^−1^ bw for di(2-ethylhexyl) phthalate (DEHP), 0.50 mg × kg^−1^ bw for benzylbutyl phthalate (BBP), and a group TDI of 0.15 mg × kg^−1^ bw for diisodecyl phthalate (DiDP) and diisononyl phthalate (DiNP) (22–26). Russia has also established the rules governing the packaging components migration tests using simulators of food (Technical Regulations CU TR 005/2011 on the safety of the packaging). However, it is still inadequately controlled. Although there were many studies on phthalates in packaging materials and food in Europe and America (27–31), Russia lacks information. Hence, this study aimed to evaluate the migration of phthalates from various common packaging materials to beef sold in the Russian meat market.

## Materials and Methods

### Standards, Solvents, and Chemicals

Phthalate standards, including dimethyl terephthalate (DMTP), di-n-butyl phthalate (DnBP), and diisooctyl phthalate (DiOP), were procured from ChromLab Ltd. (Lyubertsy, Russia). LC grade or glass-distilled solvents were used. Dichloromethane and hexane were from Merck (Darmstadt, Germany). HPLC gradient grade methanol was from J.T.Baker (Deventer, Netherlands). Water is produced using Milli-Q Type 1 Ultrapure Water Systems (Millipore, Burlington, Massachusetts, United States).

Standard stock solution for each analyte (DMTP, DnBP, and DiOP) was prepared in methanol at a 1 mg·ml^−1^ concentration. These solutions were further diluted in methanol, yielding various concentrations (0.005, 0.01, 0.1, and 0.5 mg·ml^−1^) for constructing the calibration curves. All working solutions are prepared freshly before use.

During all manipulations, contact with any plastic material was not allowed. All glassware and accessories were rinsed with methanol, acetone, and n-hexane, immediately before and after use.

### Sample Collection

Beef striploin (50 samples) and packaging materials (35 samples) were secured from various shopping areas in Saint-Petersburg and Leningrad region, Northwestern Federal District, Russia, between November 2020 and May 2021. Samples of beef striploin were selected with an average fat content of about 20%. It should be noted that the distribution networks of the Northwestern Federal District and the range of goods are common or very similar for beef and packaging materials compared to most regions of Russia.

Farm beef samples (40 samples) were obtained from striploin cuts weighing 4.5–5 kg each from three carcasses. These three striploin cuts were delivered to the laboratory in glass containers. In the laboratory, the cuts were carved into appropriate pieces and analyzed, minced and/or packed in five samples for each type of processing. Each sample was packaged and sealed in selected packaging material, as shown in Table 1. Each packaging sample was taken from a single roll or unit of packaging material. Samples of commercially packaged beef were purchased in quantities of five for each of the two types. The pH of the farm fresh beef was 5.7 ± 0.1 (24 h), increased to 6.1 ± 0.1 (120 h) after slaughter. The pH of the chilled commercially packaged beef samples was 6.0 ± 0.2. The water content of farm fresh beef, commercially packaged beef sample 1, and commercially packaged beef sample two were 64.5 ± 0.8, 65.7 ± 0.5, and 65.2 ± 0.7, whereas the fat contents were 16 ± 1, 17.3 ± 0.8, and 18.6 ± 0.7, respectively. Except for commercially packaged and minced beef, all samples were sliced at 10 ± 2 mm thickness. Some samples were subjected to microwave heat treatment for 10 min at a power of 900 W. Each sample was homogenized in a stainless-steel laboratory blender Grindomix GM200 (Retsch GmbH, Haan, Germany) before analysis. The blender was thoroughly cleaned after processing each sample to avoid cross-contamination.

**Table 1:** Food samples, packaging types, and conditions.

**N**	**Food**	**Packaging and conditions**	**Type of plastic**
1	Farm fresh beef	Obtained at a farm right after slaughter and placed in a glass container	–
2	Chilled/minced farm beef	Cut or minced from the farm-fresh beef, vacuum sealed and stored at 2°C for 7 days	PET/PE^a^
3	Farm frozen beef	Cuts from farm-fresh beef, vacuum sealed and stored at −18°C for 14 days	PET/PE
4	Farm chilled beef	Cuts from farm-fresh beef, wrapped in packaging paper or a string bag, and stored at 2°C for 7 days	PAP^b^ or PP^c^
5	Farm baked beef	Cuts from farm-fresh beef, wrapped in a baking sleeve or a polyethylene film, subjected to microwave heat treatment and stored at 2°C for 7 days	PET^d^ or LDPEs^e^
6	Chilled beef sample 1	Obtained from a shop–commercially packaged in a polyethylene bag	LDPE
7	Chilled beef sample 2	Obtained from a shop–commercially packaged using vacuum packaging	PET/PE

a *Polyethylene terephthalate/Polyethylene*.

b *Paper*.

c *Polypropylene*.

d *Polyethylene terephthalate*.

e *Low-density polyethylene*.

### Extraction

To ensure low backgrounds from phthalate contamination, all glassware was rinsed with glass-distilled acetone immediately before use and only glass-distilled or HPLC grade solvents were utilized. At all stages of analysis, contact between food samples and materials other than glass, PTFE, or stainless steel was avoided. Extraction using organic solvents is widely used for phthalates analysis in solid matrix samples, such as food-packaging materials and foods (32). An average sample with a mass of about 10 g was taken from each packaged striploin or minced beef. Average meat samples were blended with the same mass of sodium sulfate and 50 ml of dichloromethane. The supernatant was filtered and removed by rotary evaporation. The weight of extracted fat was determined. Plasticizers were then isolated from aliquots of the lipid material with hexane, then analyzed by gas chromatography coupled with mass spectrometry (GC-MS).

### Gas Chromatography

**Figure 1:**
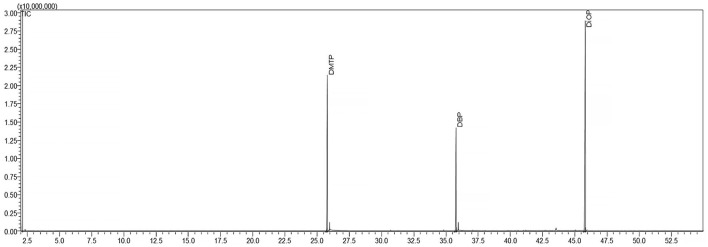
GC–MS chromatograms of standard methanol solutions of DMTP, DnBP, and DiOP in SCAN mode.

Analysis was carried out by GC-2010 Plus gas chromatograph (Shimadzu, Kyoto, Japan) linked with gas chromatograph-mass spectrometer GCMS-TQ8040. The separation was performed on a cross bonded a 30 m × 0.25 mm I.D. Rxi-5Sil MS column (Restek, Bellefonte, Pennsylvania, USA) coated with 5% diphenyl 95% dimethylpolysiloxane (film thickness 0.25 μm). The oven temperature was programmed from 50°C (holding time 5 min) to 300°C at 5°C min^−1^. One μl was injected in the injection mode (split ratio 1:10). Helium was used as the carrier gas (1.1 ml min−1), and the injection temperature was set at 250°C. The electron-impact ionization mass spectrometer was operated as follows: ionization voltage, 70 eV; interface and ion source temperatures were 275°C and 200°C, respectively; scan mode, mass range 40.0–500.0; solvent cut time 2 min. Plasticizer identification was performed by comparing mass spectra with those in the mass spectral library (NIST, Gaithersburg, USA). The phthalates were detected using a SCAN mode (Figure 1).

A base peak and a qualifier ion were chosen by the intensity of signals for each phthalate (Table 2). The dominant product ion 149 *m/z* was used to quantify all phthalates, except for DMTP. The ion 163 *m/z* was used for DMTP quantification. The qualifier to target the ion ratio of each phthalate had to be <20% of the same in a standard solution for positive identification. The retention times on the Rxi-5Sil MS column, similarity range and the characteristic *m/z* values for these compounds are summarized in Table 2.

**Table 2:** Conditions for GC-MS analysis of phthalates.

**N**	**Phthalate compound**	**Retention time (min)**	**Base peak (m/z)**	**Qualifier ion (m/z)**	**Similarity (%)**
1	DMTP	25.7 ± 0.2	163	194	96–98
2	DnBP	35.7 ± 0.2	149	278	93–96
3	DiOP	45.8 ± 0.3	149	390	90–95

Quantification was based on comparing the areas of identified peaks with calibration curves in GC-MS Solution software (Shimadzu, Kyoto, Japan). In the studied concentration range, the calibration curves fitted a linear mode (*R*^2^> 0.99). The State Pharmacopeia of the Russian Federation (XIV edition, 2018) indicates that in most cases, linear models are used if they meet the condition *R* ≥ 0.99. In our case, for the least-squares model, this corresponds to *R*^2^ ≥ 0.9801. Also, *R*^2^> 0.99 corresponds to the previously developed methods for determining phthalates in meat (33). Method performance is shown in Table 3.

**Table 3:** Method performance for GC-MS analysis of phthalates.

**N**	**Phthalate compound**	**Recovery (%)**	**RSD (%)**	**LOD (μg ·kg^**−1**^)**	**LOQ (μg ·kg^**−1**^)**
1	DMTP	94	9	1	7
2	DnBP	93	7	2	7
3	DiOP	93	10	3	10

To minimize the risk of contamination, each analytical batch was composed of several samples of blank solvent, standard calibration solutions, a reference, and several experimental samples (five replicates at the most). The whole procedure was carried out using minimum solvents and glassware and as fast and straightforward as possible. Mathematical statistics methods were used for data processing at a theoretical frequency of 0.95. To determine the significant differences between the values, a two-sample *t*-test was used for each pair of means.

## Results and Discussion

Fasano et al. (11) investigated the migration of phthalates from a common form of food packaging. They concluded that simulants may be helpful for identification and comparing legislated maximum concentrations of compounds migrating from containers. However, this is not always adequate to real conditions, and lately, migration experiments should be performed directly in food. The use of mass spectrometry makes it possible to evaluate the migration of phthalates not only in simple phantom mixtures but also in real objects with a complex matrix (34). So, we used the meat samples as research objects in our study.

**Figure 2:**
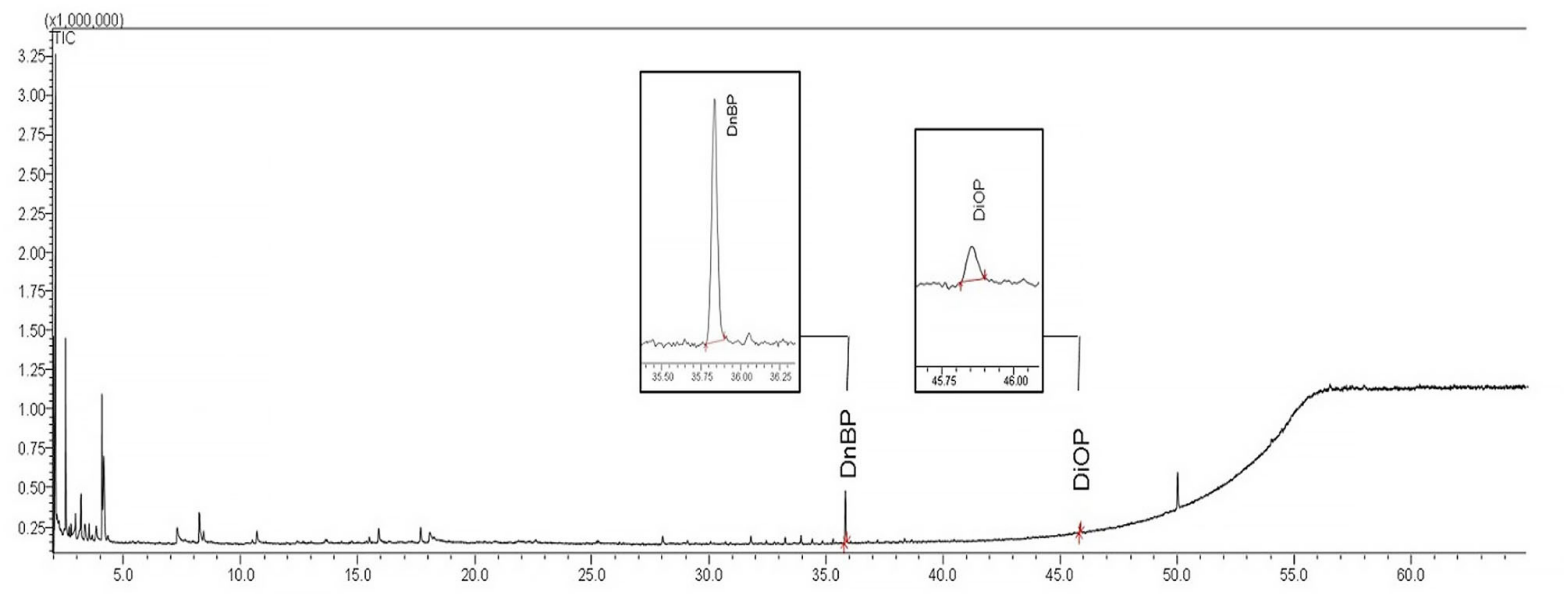
GC–MS chromatogram of an extract of chilled minced farm beef in a vacuum package.

Figure 2 shows the typical chromatogram of chilled minced farm beef extract previously stored in a vacuum package. Selected conditions gave an excellent resolution of phthalates peaks, and the matrix did not interfere with the identification. This proves that phthalates could be adequately identified and quantified in all investigated samples.

The food's specific surface area, moisture, fat content, and thermal (microwave) treating impact the degree and rate of migration of harmful substances (11). That is why cut and minced samples were prepared for the research. The effect of temperature on migration was investigated for storage at 2 and −18°C, as well as after heat treatment using microwave radiation. The results of various beef samples are presented in Table 4.

**Table 4:** Levels of phthalates in beef samples μg·kg^−1^ (mean ± sd).

**N**	**Food**	**Packaging**	**DMTP**	**DnBP**	**DiOP**
1	Farm fresh beef	–	ND^*^	35.1 ± 0.2a	ND
2	Chilled minced farm beef	Vacuum package	ND	378.5 ± 0.1b	37 ± 1s
3	Chilled farm beef	Vacuum package	ND	88.2 ± 0.5c	12.2 ± 0.1t
4	Frozen farm beef	Vacuum package	ND	89 ± 1d	10 ± 1u
5	Chilled farm beef	Paper	ND	41 ± 2e	11 ± 2t,u
6	Chilled farm beef	String bag	ND	73.2 ± 0.4f	23.2 ± 0.3v
7	Baked farm beef	Baking sleeve	ND	34.5 ± 0.7a	15.2 ± 0.4w
8	Baked farm beef	Polyethylene package	ND	75 ± 3f	24 ± 2v
9	Chilled beef sample 1	Polyethylene package	ND	38 ± 2g	10 ± 1u
10	Chilled beef sample 2	Vacuum package	ND	43.8 ± 0.8h	<10^**^

* *ND, not detected*.

** *<10 indicates that the value was lower than LOQ, but higher than LOD. Different lower-case letters indicate significant differences among samples for each phthalate (p < 0.05)*.

In general, the obtained values of phthalate concentrations vary wildly, even for the same type of plastic. This might be attributed to the fact that each plastic material uses different plasticizers and additives.

We have found phthalates in the farm-fresh beef that has been collected directly after the slaughter at the farm with no contact with any packaging materials. This finding suggests phthalates contamination of farm fodder or water, which necessitated further research.

The use of all packaging and processing options for farm fresh beef increased phthalate content compared to untreated and unpackaged samples. This indicates the migration of these xenobiotics from all types of the investigated packaging for almost any condition. The large contact area and the presence of more fat on the surface of the minced meat resulted in significantly higher phthalate migration (DnBP: 378.5 μg·kg^−1^, DiOP: 37 μg·kg^−1^) than beef slices (DnBP: 88.2 μg·kg^−1^, DiOP: 12.2 μg·kg^−1^). Deserves attention, frozen storage resulted in nearly the same phthalate levels (DnBP: 89 μg·kg^−1^, DiOP: 10 μg·kg^−1^) as refrigerated storage (DnBP: 88.2 μg·kg^−1^, DiOP: 12.2 μg·kg^−1^). This is probably due to phthalates migration during freezing and thawing since it is evident that the migration of components at −18°C should be very slow. This requires further research. However, as a primary recommendation, more intensive freezing of packaged meat products and defrosting outside plastic packaging can be proposed.

DnBP showed the highest mean values for most samples ranging from 34.5 to 378.5 μg·kg^−1^. DiOP displayed the lower mean values, ranging from LOD to 37 μg·kg^−1^. Particular attention should be paid to the fact that according to regulatory documents in Russia, DnBP presence in food is not allowed. The European Union regulation EC No. 11/2011 also states that DnBP is not allowed in fat-containing food. DiOP contents correspond to Russian normative documents (CU TR 005/2011). Also, they correspond to the European Union regulation EC No. 10/2011, which established a limit of 60 mg·kg^−1^ for DiOP in food products. Regarding phthalates regulation, the USA and the European Union have harmonized their directives and implemented a “threshold policy” (35). Thus, the conclusions drawn regarding the obtained DnBP and DiOP concentrations for the European Union are also relevant for the United States. Polyethylene terephthalate (PET) used in the study is usually made with the addition of DMTP, and it was expected to be found. However, DMTP could not be recovered and was not detected in any samples. Thus, the DMTP concentration was below the LOD in the studied samples. One of the possible reasons for this may be its less use in the production of plastic packaging since it is more used as solvent and fixative in fragrances, additive in cosmetics, medical devices, and household and personal care products (36).

Analysis of phthalates in foods sold on the Belgian market revealed DnBP in 14 out of 22 meat and meat products with concentrations of up to 15.0 μg·kg^−1^(37). These concentrations in beef correlated with predicted by environmental food transfer model for organic contaminants in Europe (5). DnBP was determined in all the analyzed samples of spices used to cook the chicken meat in concentrations ranging from 3.47 to 29.3 μg·kg^−1^ (38). The chicken meat samples roasted with spices in a plastic bag presented concentrations from 0.1 to 1.17 μg·kg^−1^ for DnBP. DnBP was the most frequently detected ortho-phthalate in USA fast food samples (39). It was detected in 81% of all food items in median concentrations of 2.4–4.8 μg·kg^−1^. A study of convenience meat products on the Chinese market (40) found a median DnBP content of 336 μg·kg^−1^ for 48 samples. Also, it was found that the migration of the phthalates into foods increased with time and the temperature and their content in the convenience foods near the shelf-life was much higher than those which were just manufactured. This study confirms these trends concerning plastic packaging available on the Russian market. Research on phthalates in food packaged in various types of plastics worldwide suggests a need to carefully analyze their overall consumption and assess the associated health risks (33, 35).

Besides polymer packages, we found phthalates in foods stored in wrapping paper. The chilled farm beef samples wrapped in paper contained DnBP and DiOP 41 and 11 μg·kg^−1^, respectively. The possible reason is the use of recycled cartons to produce paper. Using recycled fibers comes with new challenges, such as controlling potential packaging contamination by harmful chemicals introduced by using pre-and post-consumer waste. Numerous studies have revealed the migration of various contaminants, such as phthalates (41), diisopropyl naphthalenes, benzophenones and others (42). In a study conducted by USA Food and Drug Administration scientists reported detectable concentrations of DnBP in paper-based fast food packaging collected from restaurants (31). Also, this is understandable, as phthalate compounds are widely used additives for printing inks and lacquers in food contact materials.

Daily intake (DI) was determined and compared with its tolerable daily intake (TDI) value established by the European Food Safety Authority to assess the health risks of human exposure to phthalate residues in beef. The DnBP TDI value is 10 μg·kg^−1^ body weight a day (23). Since no TDI values have been established for DiOP, the risk assessment was carried out only in relation to the detected DnBP. The DI (μg·kg^−1^·day^−1^) was determined by multiplying the daily beef consumption (kg·day^−1^) by obtaining mean DnBP concentration (μg·kg^−1^) and dividing by human body weight (kg). The daily beef consumption value of 27.4 g (0.0274 kg·day^−1^) was obtained by dividing the stated annual beef consumption in Russia by 10 kg·year^−1^ by 365 (43). An average body weight of 62 kg for adults was used as a reference value (44). The results are indicated in Table 5.

**Table 5:** Estimated DI levels of DnBP residues in beef and their percentage of TDI level in adults.

**N**	**Food**	**Packaging**	**DI (μg·kg^−1^·day^**−1**^)**	**% of TDI**
1	Farm fresh beef	–	0.016	0.16
2	Chilled minced farm beef	Vacuum package	0.167	1.67
3	Chilled farm beef	Vacuum package	0.039	0.39
4	Frozen farm beef	Vacuum package	0.039	0.39
5	Chilled farm beef	Paper	0.018	0.18
6	Chilled farm beef	String bag	0.032	0.32
7	Baked farm beef	Baking sleeve	0.015	0.15
8	Baked farm beef	PE package	0.033	0.33
9	Chilled beef sample 1	PE package	0.017	0.17
10	Chilled beef sample 2	Vacuum package	0.019	0.19

Thus, the daily intake did not exceed TDI for all types of packaging and beef processing. At the same time, the largest percentage of TDI was determined for chilled minced beef in vacuum packaging. In the annual and, accordingly, the average daily beef consumption is 1.36% (10 kg per year of beef from 735.6 kg per year of all food products) (45). Thus, if we assume a similar level of phthalates translocation from plastic packaging to other food products, as well as previously unaccounted drinking water, the daily intake of DnBP and other phthalates may exceed TDI. Thus, it is necessary to reduce the amount of plastic packaging and reduce the content and migration of phthalates into food from it. Also, an obvious conclusion is a need for the global introduction of new types of packaging materials without phthalates. Bio-based resources can be a base for producing biopolymers in food packaging applications instead of conventional plastics traditionally produced from fossil fuels. Microorganisms can produce biopolymers through fermentative processes of different bioresources (e.g., polyhydroxyalkanoates), and biomass may be produced directly from different types of plants (starch, cellulose, pectin, zein, gluten) and animal materials (chitosan, gelatin, caseins) (46). Biopolymer production does not include phthalates as glyceryl monoesters, glycerol, fatty acids, and polyethylene glycol are used as common plasticizers (47).

## Conclusions

The Technical Regulations TR 005/2011 on the safety of the packaging were adopted in 2011 in the Russian Federation. This document defined allowable migration amounts for DiOP of 2.00 mg·L^−1^, and migration of DnBP into food products was not allowed. The examined food packages from the Russian market do not meet the requirements of this standard, as DnBP migrates to food products. Perhaps the manufacturers are still using the previous instruction from 1972, according to which the allowable migration amounts for DnBP was 0.25 mg·L^−1^. The obtained results require discussion by all participants of the production processes and the use and control of food packaging materials.

The most alarming results obtained in this study are the presence of phthalates in beef produced on a farm in the Leningrad region and not subjected to packaging. Presumably, phthalates could enter animals' diet along with fodder, compound feed, or water. A full-scale study is needed to determine the pathways and sources of phthalates migration in the food chain. The implementation of separate collection and recycling of plastic materials in Russia may be a way to reduce the contamination of farm animals food chains with phthalates. Moreover, this should be carried out in such a way as to avoid cross-contamination of materials, for example, recycled paper. In addition, it is necessary to strive to reduce the use of plastic packaging. At present, both the encapsulated forms of food ingredients (48) and the edible food coatings that increase food shelf-life are being developed to improve product stability during storage (49).

## Data Availability Statement

The raw data supporting the conclusions of this article will be made available by the authors, without undue reservation.

## Author Contributions

DB contributed to the study concept. DB and AE-A critically reviewed the article. DB, MB, IB, BA, and WL contributed to the design of the manuscript and figure preparation and edition. DB, MB, IB, BA, WL, and AE-A contributed to the acquisition and analysis of data and drafted the manuscript. All authors gave final approval for all aspects of the work, agreed to be fully accountable for ensuring the integrity and accuracy of the work, read, and approved the final manuscript.

## Funding

This study was supported by the Russian Science Foundation (Grant No. 21-16-00124, https://rscf.ru/en/project/21-16-00124/).

## Conflict of Interest

The reviewer (FO) declared a shared affiliation with one of the authors, (AE-A), to the handling editor at the time of review. The authors declare that the research was conducted in the absence of any commercial or financial relationships that could be construed as a potential conflict of interest.

## Publisher's Note

All claims expressed in this article are solely those of the authors and do not necessarily represent those of their affiliated organizations, or those of the publisher, the editors and the reviewers. Any product that may be evaluated in this article, or claim that may be made by its manufacturer, is not guaranteed or endorsed by the publisher.
